# Fracture of the Neck of the Femur within the Capsule—Bony Union

**Published:** 1849-12

**Authors:** 


*Fracture of the Neck of the Femur within the Capsule—Bony Union.*—Dr. Condit reports a case of fracture of the neck of the femur, in a man, aged 80. The accident happened in May, and the patient died in November following. The following are the post-mortem appearances: The muscles and other structures around the cervix femoris were more pale than usual; the capsular ligament was entire, giving no appearance of laceration. The ligamentum teres was vascular; the acetabulum was normal in appearance. The neck of the femur was shortened, and the fracture was discovered to be wholly within it.—The head of the bone had been broken across transversely, exactly where it joins the neck. The ridge, characteristic of the seat of fracture, had been thrown out, and the reunion was firm for more than three-quarters of the circumference of the bone. The limb having been drawn up by the contraction of the muscles, a considerable angle was formed by the head and neck at the point of juncture, but they were as firmly united by osseous formation as if they never were separated. On the upper side, where the fractured edges were not in apposition, union was not yet complete, but ossification was going on upon all the broken surface, and had the patient lived a few months, would doubtless have been perfected. Could the state of the injured part have been by any means ascertained, and had not the condition of the ankle forbidden it, the patient, I think, might safely have walked; there was sufficient firmness at the fracture for the limb to have contribute its share of support to the trunk. From a fear lest some accident should befal the specimen in handling it, I left it with a mechanic to have the head protected by a covering wire. He placed it for safe keeping in a desk in his room, belonging to another man, who, removing the desk in his absence, threw out the bone, supposing it to be of no value, and though diligent search was made, it was not recovered. I had the preparation in my possession for two or three years, and during that time it was shown to many members of the profession, who expressed but one opinion, that it was a case in which a complete fracture entirely within the capsule was reunited by ossification.—*American Journal of Medical Sciences.*

